# Compositional factors driving antibacterial efficacy in healthcare wet wipe products

**DOI:** 10.3389/fmicb.2025.1582630

**Published:** 2025-04-28

**Authors:** Carolina Angulo-Pineda, Jian Ren Lu, Sarah Cartmell, Andrew J. McBain

**Affiliations:** ^1^Department of Materials, School of Natural Sciences, Faculty of Science and Engineering and The Henry Royce Institute, The University of Manchester, Manchester, United Kingdom; ^2^Biological Physics Laboratory, Department of Physics and Astronomy, School of Natural Science, The University of Manchester, Manchester, United Kingdom; ^3^Division of Pharmacy and Optometry, Faculty of Biology, Medicine and Health, The University of Manchester, Manchester, United Kingdom

**Keywords:** non-woven characterization, hospital disinfection, antibacterial wet wipes, surface disinfection, disinfectant products

## Abstract

Healthcare-associated infections represent a persistent global pathogenic challenge, with surface contamination serving as a critical transmission vector. Antibacterial wet wipes are widely used in public health service settings, but their effectiveness varies depending on their composition and design. Evaluating existing products based on objective criteria provides a valuable foundation for enhancing future functional wet wipe formulations. In this study, four compositionally distinct proprietary antimicrobial wet wipes were assessed using objective criteria to provide a foundation for designing functional and biodegradable wet wipe formulations with enhanced properties. The mechanical properties, chemical composition, surface tension, disinfectant release, and morphological structure of four widely used wet wipe products in UK and European healthcare settings were evaluated. These products are anonymised as HP (containing oxidizing agents), BDB, DPA, and ADM (based on quaternary ammonium compounds). Antibacterial performance was tested according to EN 16615, EN 13727, and ASTM E2967 standards (suspension and wiperator-based methods) using contact times of 30 and 60 s, against *E. coli*, *S. aureus*, MRSA, and *A. baumannii*. The BDB product underperformed in the analysis based on the EN 16615 standard, particularly at the short contact time, where it was ineffective against *E. coli* and *S. aureus*. In contrast, HPE, DPA, and ADM products demonstrated high efficiency, achieving >5 Log bacterial reduction within 60 s for all tested strains. The ADM product demonstrated superior antibacterial performance, eliminating *A. baumannii* in the shortest contact time and avoiding spread of bacteria to clean surfaces. Our findings demonstrate a correlation between the liquid release rate of the wipe and the biocidal agents used, with effectiveness in reducing bacterial viability on surfaces. This research establishes a framework for optimizing disinfectant wipe design by emphasizing the importance of material properties and disinfectant formulation in determining overall effectiveness.

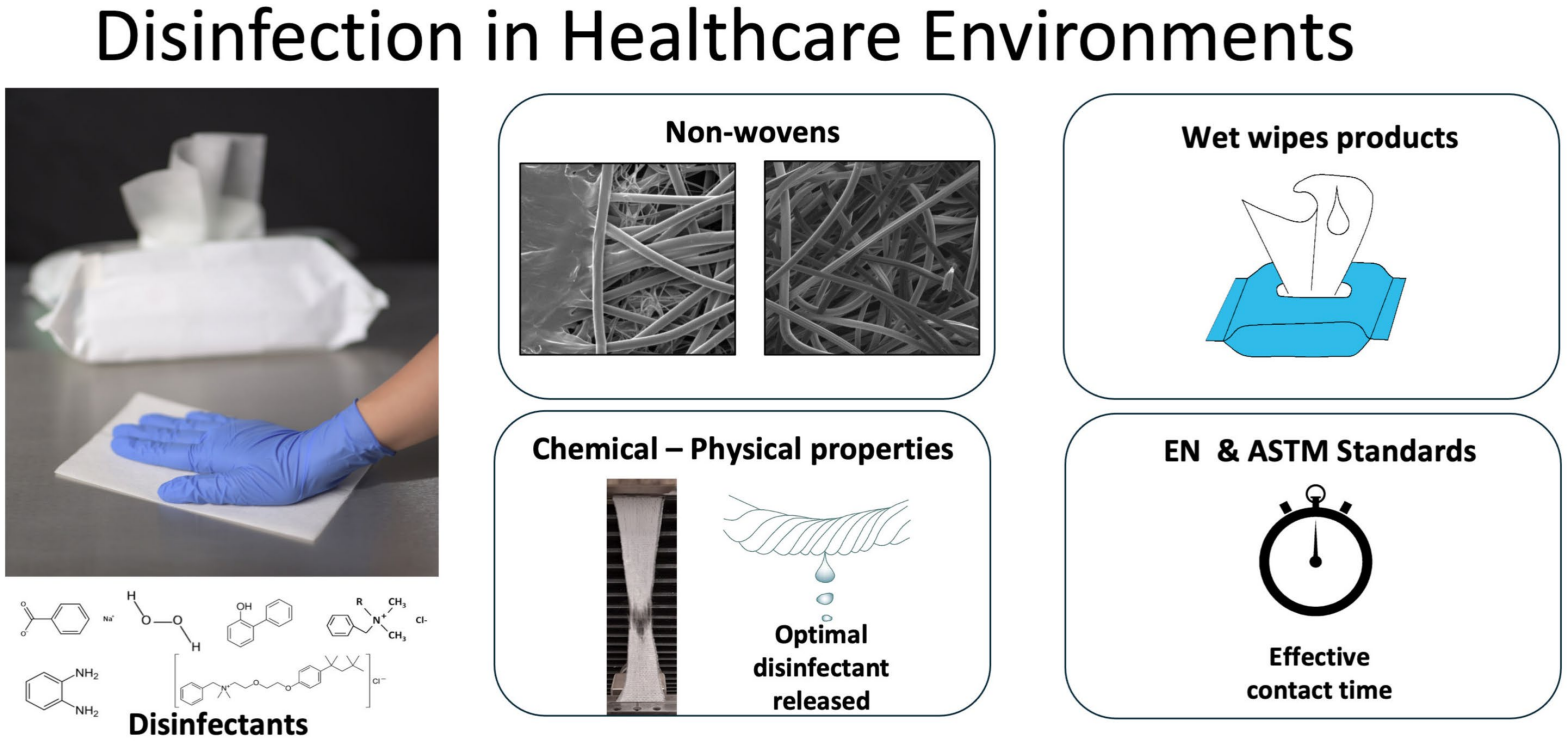

## Introduction

Pathogenic microorganisms present in common environments, such as public transport, schools and hospitals, among others are an important threat to human health. These pathogens may cause infections when entering the human body via different pathways (Dalton et al., 2020). The colonization of skin surfaces is a quick route for transfer from hands to mucosa, mouth or nose, and wound sites. One of the most critical environments in this respect are hospitals and central healthcare services (Alfa et al., 2015). Surfaces such as floors, walls, bed rails, light switch and mattresses have been identified as sources of contamination (Huslage et al., 2010).

The widespread and persistent use of antibiotics, coupled with increased exposure to pathogens in healthcare environments, has significantly contributed to the emergence of multidrug-resistant (MDR) bacteria. Of particular concern are the ESKAPE pathogens, which include *Enterococcus faecium*, *Staphylococcus aureus*, including methicillin-resistant *S. aureus* (MRSA), *Klebsiella pneumoniae*, *Acinetobacter baumannii*, *Pseudomonas aeruginosa*, and *Enterobacter* species (Panda et al., 2022). These organisms are now responsible for a substantial proportion of Hospital-Acquired Infections (HAIs), contributing significantly to the global burden of nosocomial infections.

The challenge of managing these infections is further exacerbated by the ability of some pathogens, such as *A. baumannii* and MRSA, to persist on hospital surfaces for extended periods, ranging from days to weeks (Kramer et al., 2006). This prolonged environmental survival not only facilitates the spread of these organisms but also complicates infection control efforts in healthcare settings. Thus, the transfer of nosocomial pathogens may occur from high-touch environmental surfaces and medical devices between patients, visitors, and healthcare workers (Weber et al., 2013). This health issue brings significantly increased patient mortality and raises the economic costs of hospitalization and medical treatments (Collins, 2008).

Effective infection control in hospitals involves a combination of strategies, including proper hand hygiene, regular surface disinfection, and the use of antimicrobial materials. These strategies collectively play a vital role in minimizing HAIs and antimicrobial resistance (Haque et al., 2020). Surface cleaning can reduce microbial loads and their dissemination (Browne et al., 2023). It has been demonstrated that a periodic routine of disinfection of environmental surfaces can decrease the risk of patients developing infections (Song et al., 2022).

Wet wipes for healthcare applications offer an effective solution for efficacious infection prevention via an effective disinfection routines (Boyce, 2021). Commercial wet wipe products intended to eliminate microorganisms on surfaces, are available; however, there are certain issues related to their design and performance. These include concerns about the efficacy and efficiency of antimicrobial agents utilized, as well as their safety for both skin contact and other surfaces.

Most currently available wet wipes are non-biodegradable. Biodegradable wet wipes are designed to break down naturally over time, reducing environmental impact. There is an environmental concern associated with their use, due to residues that must be dispersed and carbon dioxide emitted (Shruti et al., 2021). These residues often include synthetic fibers, and chemical additives that are not easily degradable, contributing to landfill overflow and marine pollution. Improper disposal, such as flushing wet wipes, exacerbates issues like sewer blockages and microplastic contamination in aquatic environments (Carr et al., 2016). In the UK, this concern has also been acknowledged within the public health sector. The National Health Service (NHS) for example, is committed to incorporate environmentally conscious methods in providing healthcare services with the aim of reducing CO_2_ emissions by 80% between 2028 and 2032 (Watts et al., 2022). To meet this requirement, it is important to explore sustainable alternatives that can replace single-use plastics or fibers and mitigate environmental pollution. Recently, plastic-free fibers from natural sources have been used in cleaning products, however, challenges remain in their design and effectiveness for disinfecting healthcare facilities due to the chemical–physical incompatibility between fibers and liquid disinfectants, as well as the precise application of an optimal amount of antimicrobial liquid (Song et al., 2022; Yun et al., 2020). Therefore, it is important to understand surface properties of natural-based fibers to obtain a systematic assessment of their antimicrobial performance. The excellent antimicrobial efficiency of plastic-based fibers has been partly attributed to the physiochemical binding of such as quaternary ammonium compounds (Quats), with fibers (Boyce et al., 2015; Pascoe et al., 2022). These compounds are widely used in hospitals and healthcare settings to control hospital-acquired infections. Biocidal agents, which include antiseptics and disinfectants, have a broader range of activity and their applications differ from antibiotics. Unlike antibiotics that target specific sites inside cells, biocides mainly target microbial membranes and inactivate microorganisms disrupting cellular membranes (Maillard and Pascoe, 2024). Interactions between biocides and bacterial targets depend not only on chemical composition of biocides, but also by a variety of other factors that are relevant to their practical applications (Maillard et al., 2013). Inadequate understanding among manufacturers regarding the diverse chemical compositions of biocides and their influence on antimicrobial efficacy, when coupled with improper utilization, inaccurate dilution, or insufficient contact duration, may induce microbial susceptibility or persistence and even potentially facilitate the evolution of resistance. This resistance may extend to unrelated compounds like antibiotics, (Maillard and Pascoe, 2024).

Most wet wipes currently used in hospital and healthcare environments for disinfection are made of non-biodegradable plastic fibers with quaternary ammonium compound-based formulations. The effectiveness of plastic fibers can be partly attributed to both the volume and concentration of liquid disinfectant released during the wiping process (Song et al., 2022). This is supported by the ability of the biocide to meet antimicrobial performance standards, such as those outlined by British standard institue (BS) and European (EN) protocols for assessing products intended for clinical applications; it is also evidenced by the biocide’s capacity to satisfy antimicrobial performance criteria, such as the standards established by BS EN standards for evaluating products intended for clinical applications (Wesgate et al., 2018).

To accurately evaluate antimicrobial effectiveness, factors such as product design, formulation, organic load, material surface properties, interactions with biocides, temperature changes, dilution levels, and testing methods must be carefully considered.

Several tests can be used to evaluate the efficacy of wet wipes, depending the intended application and specific microorganisms that must be eradicated (Klarczyk et al., 2023; Tyski et al., 2021). Commercial brands of healthcare product should meet the BS EN standards compilated in BS EN14885:2022 to ensure quality and antimicrobial efficacy of disinfectant products. The BS EN 13727 standard (CEN, 2015b) is widely adopted to evaluate the efficacy of disinfectants against a wide range of bacteria and yeasts in medical area. Standards such as ASTM E2967 (ASTM International, 2015) and BS EN 16615 (CEN, 2015a) are also used to evaluate hospital disinfectant wet wipes. These standards are widely adopted and ensure reproducible testing outcomes, allowing for comparisons between market products and demonstrating their effectiveness in removing harmful microorganisms in hospital environments (Jacobshagen et al., 2020; Sattar et al., 2015). These standardized protocols serve as quantitative tests to evaluate the efficacy of different wet wipes in reducing microbial contamination on surfaces in healthcare settings. All biocidal products must receive authorization before being placed on the market, and their active substances must be approved in advance. This requirement is governed by the Biocidal Products Regulation (BPR, Regulation (EU) 528/2012), which regulates the use and market placement of biocidal products. These products are used to protect humans, animals, materials, or articles from harmful organisms such as pests or bacteria through the action of their active substances. The regulation aims to harmonize the biocidal products market within the European Union while ensuring a high level of protection for human health and the environment (European Commission, 2025; European Chemicals Agency, 2025).

Despite the critical role of cleaning and disinfection in healthcare environments, a comprehensive comparative analysis of leading commercial wet wipes remains lacking. In this study, commercial products in the UK for cleaning and disinfecting surfaces within hospital environments were analyzed using BS EN and ASTM standards. Product assessments for healthcare disinfection were performed according to EN 13727 and EN 16615. Furthermore, to comprehensively evaluate disinfection effectiveness, the commercial products underwent analysis utilizing the Wiperator™ device test under the ASTM E2967 standard. This research establishes criteria for current product performance and provides a foundation for developing enhanced formulations and innovative disinfectant solutions for healthcare settings.

## Materials and methods

Four leading and widely used disinfection wipe products intended for healthcare settings, anonymized and labeled as HPE, BDB, DPA, and ADM, were utilized for testing and analysis. HPE, BDB, and DPA has a Medical Device registration (CE), otherwise all biocides actives are registered in the BPR, Regulation (EU) 528/2012. The focus was placed on evaluating the chemical composition of their fibers, only two out of the four brands openly disclose the use of polypropylene fibers which are non-biodegradable. Therefore, it can be assumed that non-woven materials used in these wipes are made from non-biodegradable polyester fibers based on evidence presented in the results section. The specific biocidal active substances within the formulation of each product are delineated in Table 1 below. Once opened, all packages have a period after open of up to 2 months. All products were used within this timeframe. To ensure the safety and quality of the products, each item was stored under laboratory conditions (room temperature and standard humidity). Additionally, to maintain product integrity, each package was sealed with a protective film and stored in a sealed bag after use.

**Table 1:** Summary of liquid disinfectant products with their main components specified.

Name	Composition of active ingredients	Fibers materials and manufacturing processes*
HPE	Hydrogen peroxide (<1% w/v, CAS-No.:7722-84-1)	Polypropylene—melting bonding
BDB	Benzalkonium chloride (<0.6% w/v CAS-No.:68424-85-1), Didecyldimethyl ammonium chloride < 0.6% w/v CAS-No.: 7173-51-5, Biphenyl-2-ol (<0.1% w/v CAS-No.: 90-43-7)	Polypropylene—melting bonding
DPA	Didecyldimethyl Ammonium Chloride (<5% w/v CAS-No.: 7173-51-5), Phosphoric acid (<0.25 w/v CAS-No.: 7664-38-2), 2-Amioethanol < 5% w/v CAS-No.: 141-43-5	Polyester—spunlace process
ADM	Alkyl(C12-16) dimethylbenzyl ammonium chloride (<2% w/v CAS-No.:68424-85-1), Didecyldimethyl ammonium chloride (<2% w/v CAS-No.: 7173-51-5), N-(3-aminopropyl)-N-docecylpropane-1,3-diamine <1% w/v CAS-No.:2372-82-9	Polypropylene—melting bonding

*Based in morphology confirmation by SEM and some brands information. Wet wipes also contain various excipients.

### Material characterization

#### Mechanical properties

Tensile tests were conducted on non-woven fibers according to the ISO 9073-3:2023 standard (International Standard, 2023). Test samples were prepared both in the machine direction (MD) and the cross-machine direction (CD), with a test specimen width of 25 mm and a constant extension rate 100 mm/min was applied during the testing process (International Standard, 2023). It was prepared fresh 21 samples per each type of commercial product and for accomplish MD and CD fiber direction, that is 42 wet wipes per each commercial sample. Using a sealing packaging was possible to keep the wet conditions of the samples.

#### Morphological analysis

Samples were prepared using a sputter coater (Quorum, Q 150 T ES/plus) to deposit onto samples 15 nm of Au/Pd electrical conductive coating. Samples were observed using SEM equipment (TESCAN VEGA 3). The diameter of non-biodegradable fibers was determined using Image J software from three separate samples (*N* = 3), with each type of wet wipe product analyzed in triplicate (*n* = 3).

#### Disinfectant liquid release from wet wipes

Using the procedures of wiping surfaces, disks, and PVC surfaces, the weights of wipes were recorded after use. These experiments were performed without bacterial inoculum with 7 different wet wipes per sample type (Jacobshagen et al., 2020).

#### Surface tension measurements

Surface tension was measured using a force tensiometer K100 by Kruss, employing probe PL01/PLC01. The vessel used was SV10 glass, diameter of 50 mm, containing a volume of 40 mL of liquid disinfectant. All experiments were conducted at a temperature of 25°C using Peltier temperature control. The liquid used was obtained from eluted disinfectant extracted from wet wipes products.

### Antibacterial evaluations

#### Bacterial strain, medium, soil load, growth conditions and contact time

To evaluate antibacterial properties, bacterial strains used in the study comprised *Escherichia coli* (ATCC 25922) (*E. coli*), *Staphylococcus aureus* (ATCC 6538), *A. baumannii* (NCTC 12156), MRSA (ATCC 43300). The bacterial cultures were incubated at 36 ± 1°C for 18 h in 10 mL of tryptic soy broth (TSB) from Merck (Jacobshagen et al., 2020; Sattar et al., 2015). Subsequently, the cultures were centrifuged at 3,000 g for 15 min, and resulting pellets were re-suspended in 5 mL of tryptone-sodium chloride (TSC) solution, prepared with 1 g of tryptone and 8.5 g of NaCl from Merck in 1 L of double-distilled water.

Bacterial suspensions were prepared following BS EN and ASTM standard procedures. To achieve this, a calibration curve was performed at 620 nm, and a test suspension verification was conducted for each test performed. The bacterial concentration range used was from 1.5 to 3 × 10^9^ (cell/mL). Recovery of viable bacteria from both control and test samples, following the specified BS EN and ASTM standards, was conducted using Tryptic Soy Agar (TSA) plates, from Formedium. Tryptone-soy-chloride (TSC) enriched with 3 g/L of bovine serum albumin (BSA) from Sigma Aldrich was used as an interference substance. Soil load was sterilized using a membrane filtration apparatus, employing a 25 mm PES filter with a 0.22 μm pore size, assembled on a syringe. The sterilized aliquots were stored at 4°C for up to 1 week. All assessments were conducted following (CEN, 2015a; CEN, 2015b; ASTM International, 2015) standards. The contact times used in this study combine the minimum contact time specified in standards with the most commonly used contact times for commercial products in real healthcare settings across Europe and the UK (Sattar et al., 2015). These contact times were 30 and 60 s.

#### Quantitative suspension testing of wet wipe eluted liquid for bactericidal activity in medical environments

**Figure 1:**
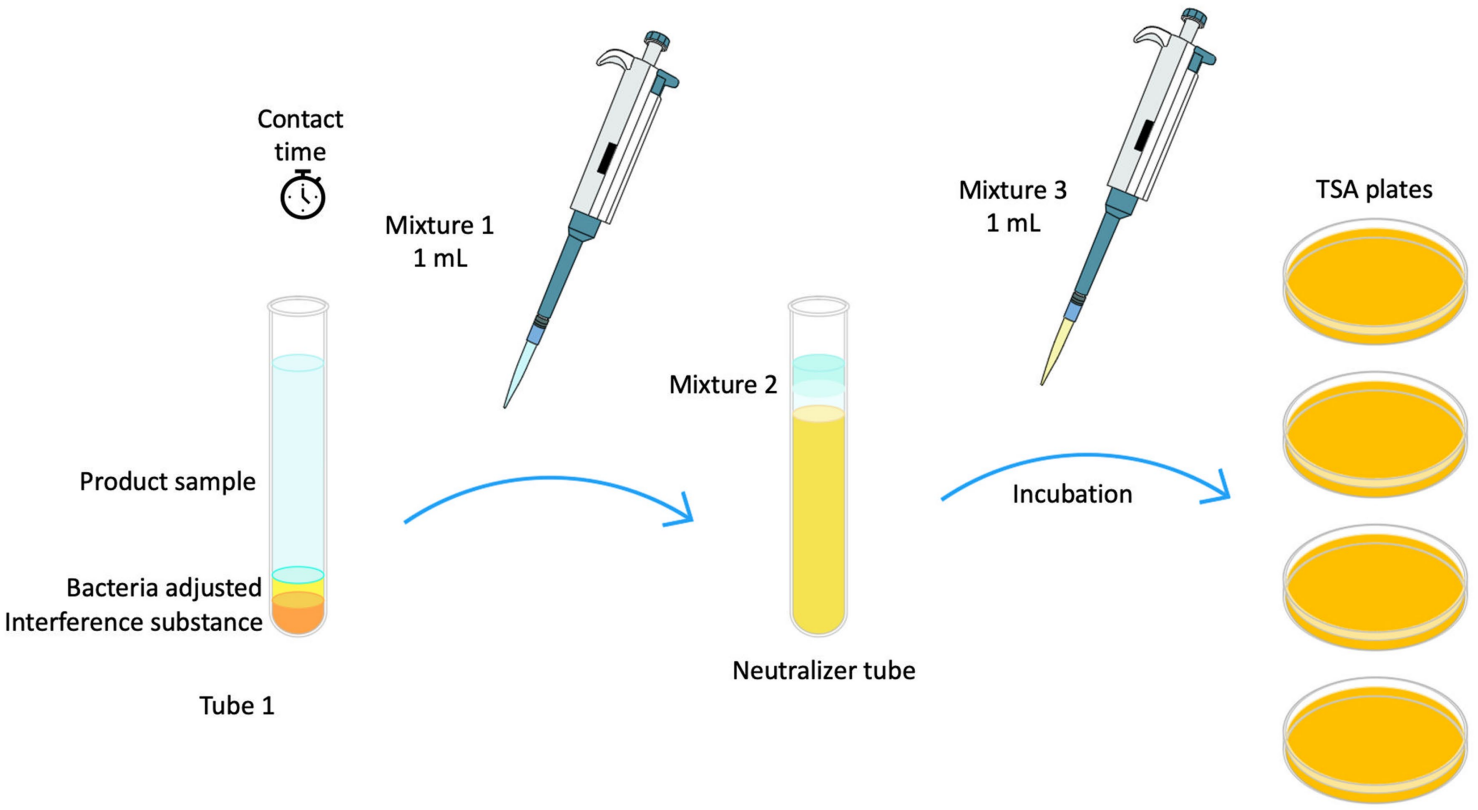
Schematic diagram illustrating EN 13727 methodology used for testing liquid disinfectant extracted from wet wipes.

Disinfectant liquid was obtained by squeezing from wet wipes. Fresh wipe eluate samples were used to perform antibacterial tests following the BS EN 13727 standard protocol (CEN, 2015b; Figure 1). Neutralizer solutions were prepared depending on type of biocide. Polysorbate 80 at a concentration of 30 g/L (Sigma Aldrich), saponin at 30 g/L (Merck), and lecithin at 3 g/L (Merck) were employed for Quat-based disinfectants. For oxidizing disinfectants, catalase at 0.25 g/L (Sigma Aldrich) with polysorbate 80 50 g/L (Sigma Aldrich) and lecithin 10 g/L (Merck) was used. Tests were performed using 60 and 30 s of contact time (Farukh et al., 2012).

#### Quantitative test method for evaluation of bactericidal activity on non-porous surfaces with mechanical action employing wipes in the medical area (4-field test)

**Figure 2:**
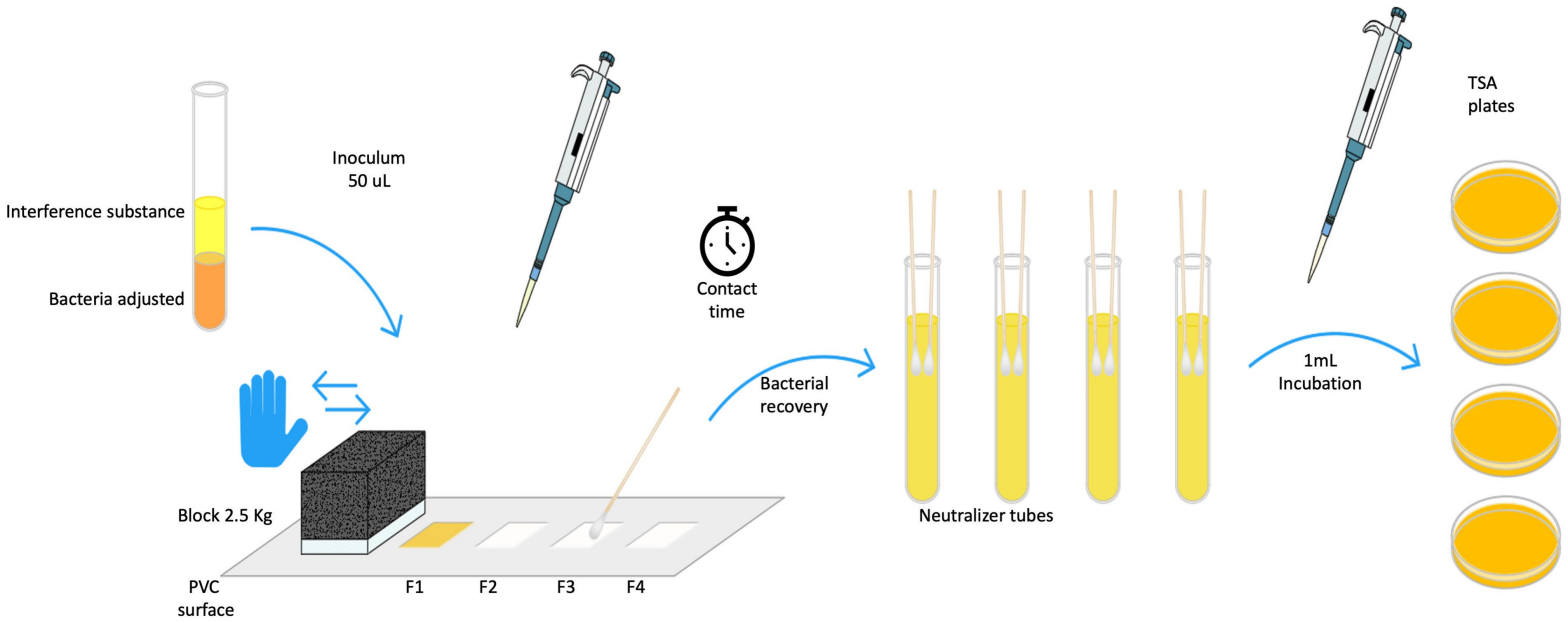
EN 16615 methodology for testing wet wipe products: Rapidly moved to and from fields 1–4 (F1 to F4) and back in less than 3 s.

Tests were carried out in accordance with the standard BS EN 16615 (CEN, 2015a), four fields of 25 cm^2^ each were delimited onto polyvinyl chloride (PVC), (40 × 50 cm, 2 mm thickness). The fields were systematically assessed for bacterial recovery after surface wiping, progressing from field 1, F1, (inoculated field) through field 2 to field 4. Fifty microliter of bacteria adjusted in clean conditions was inoculated in the center of field (F1) and uniformly spread using a sterile L-shaped spread. After 30 min the bacteria inoculum was dry and ready to perform the test. Using a unitary mass (block of 2.5 Kg) the PVC surface was wiped using an unfolded wipe as demonstrated in Figure 2.

After the contact times of 30 and 60 s, the bacterial recovery step with sterile cotton swabs was carried out. The fields were systematically rubber for bacterial recovery moving sequentially from **Field 1 (F1)**, the initially inoculated area, through **Field 2 (2)** and continuing to **Field 3 (F3)** and **Field 4**. The swabs were rinsed in appropriate neutralizer solution and then used to perform the recovery procedure across the fields, collecting bacteria after the wiping action on each surface (CEN, 2015a).

Spread plating was used to quantify bacterial reduction. Bacterial suspension, dry and recovery controls were obtained regarding the BS EN standard. Bacterial reduction and bacterial spread were evaluated from dirty field (F1) to clean fields or uninoculated (F2, F3 and F4) after wiping the surface with wet wipes products regarding criteria of standard, as seen in Figure 2.

#### Wiperator test procedure- ASTM E2967-15 standard

**Figure 3:**
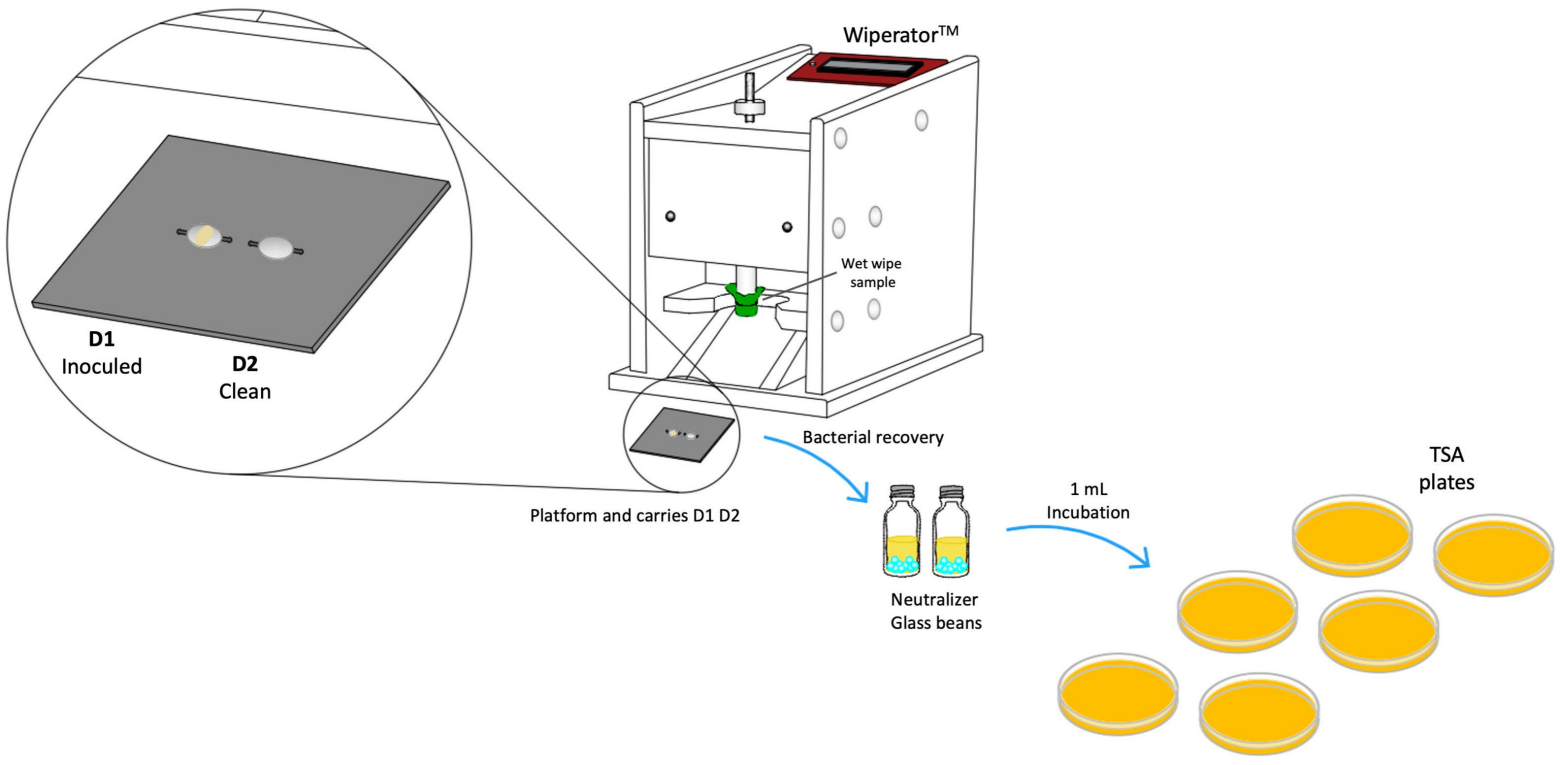
A schematic diagram illustrating the Wiperator™ device test procedures used for testing wet wipe products according to ASTM E2967-15.

The effectiveness of wipes was assessed utilizing a Wiperator™ device (FitaFlex Ltd., Canada), following procedures outlined in ASTM 2967-15 (ASTM International, 2015). Stainless-steel disk (carriers), bosses, and platforms were sterilized using an autoclave. It was inoculated a drop of adjusted bacteria (50 μL) and transferred to Petri plate to a 36 ± 1°C incubator for 30 min to dry the inoculum drop. Wet wipe packaging was inverted once for 15 s to uniformly wet its contents (Jacobshagen et al., 2020). The first two wet wipes were discarded, and 4 × 4 cm samples were prepared from different wet wipes in triplicate using a sterile scissor to obtain square from the center of each wet wipe. To prevent contamination, the cut sample tests were placed in a Petri dish for safekeeping before use in the experiment (ASTM International, 2015). The unfolded wipe samples were mounted wrapping the Teflon boss and secured using O-rings provided by the Wiperator supplier, as seen in Figure 3. Using the carrier platform with disk 1 (inocula) and disk 2 (clean) were wiped using an orbital motion of 5 s per disk with a load settled of 150 g. After wiping process and contact time of 60 s, disk 2 was wiping with the wet wipe just used. After contact time was selected (60 s), both disks were removed from platform carrier and followed sample procedure of neutralization process, disks were placed in a McCartney bottle with 1 g glass beads and 1 mL neutralizer solution (see Figure 3). Then, bottles were vortexed for 60 s. Serial dilution and Miles-Misra plating technique to inoculate appropriate dilutions onto TSA plates were performed (ASTM International, 2015). The same procedure was conducted for the control disks to quantify the recovery of bacteria colony-forming units (CFU) without disinfectant wet wipes use or mechanical action.

### Statistical analysis and analysis of results

All experiments were performed in triplicate with at least 2 independent experiments of each one. Data were analyzed according to ASTM and EN standards using GraphPad Prism® (version 10.3.1). One-way or two-way analysis of variance (ANOVA) was applied based on the number of independent variables considered.

## Results

### Morphology characterization

**Figure 4:**
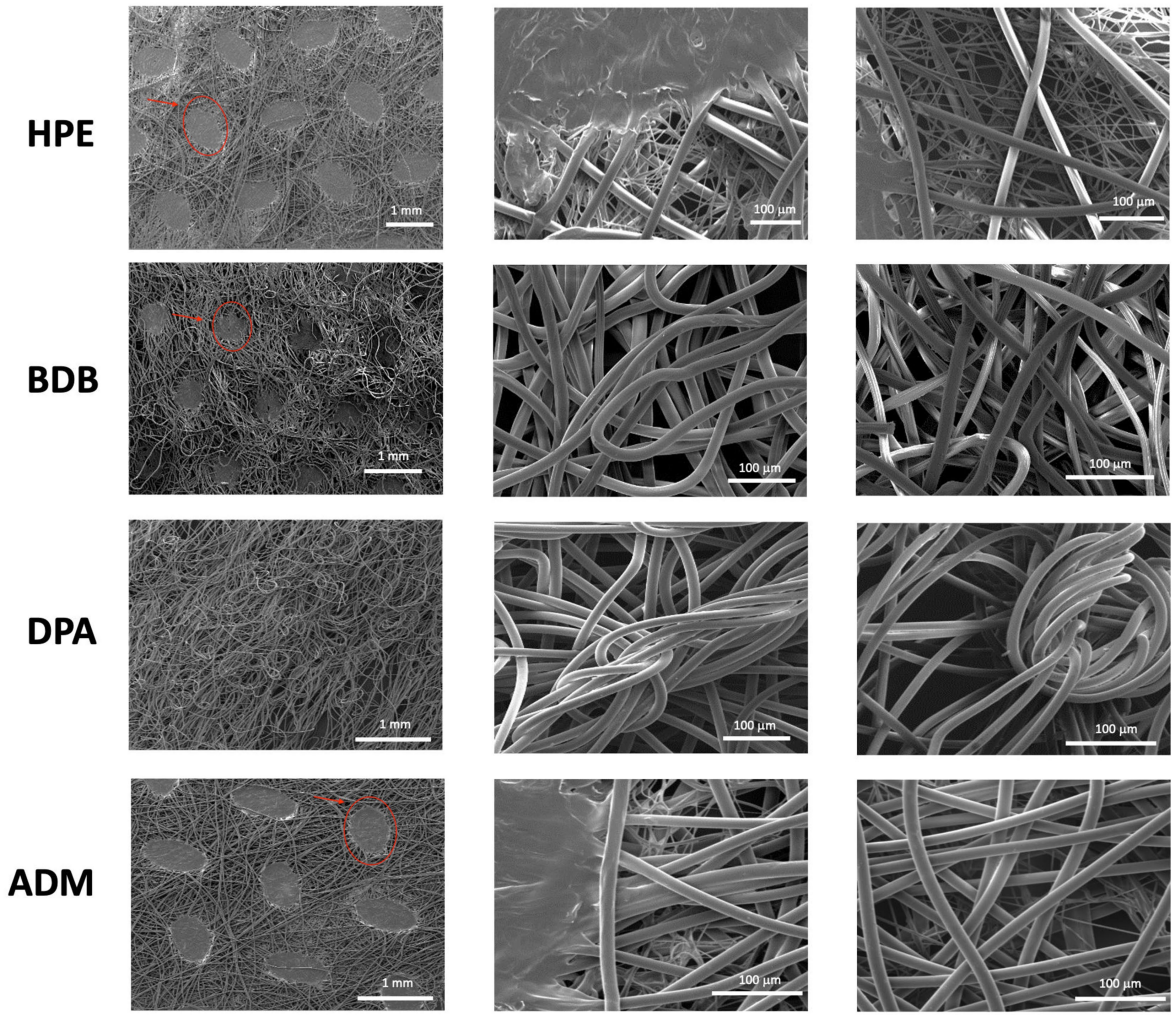
Images were obtained from three independent samples at 50× and 600× (*N* = 3), with three measurements taken in triplicate from different zones of each sample (*n* = 3). The bonding points of PP fibers are indicated by red ovals and arrows in the images.

SEM analysis was conducted on commercial fiber sheets to examine their morphological features. Non-biodegradable polyester fiber samples showed typical characteristics of thermally bonded nonwovens. Nonwoven fibers with thermally bonded patterns are widely employed and it is cost-effective to manufacture them (Farukh et al., 2012). HPE, BDB, and ADM products exhibited the two-phase structures of the PP fibers. The matrix contained obvious bonding points within the PP fiber sheet, as highlighted by the oval circles and arrows in Figure 4. The effect of the bonding points on the mechanical properties of the polyester nonwoven materials is crucial (Leucker and Schubert, 2020). These bonding points contribute to the strength and stress–strain behavior of the polyester fibers in nonwoven samples. The presence of these binding points or regions generates strong connections between fibers, enhancing their stability and durability (Hou et al., 2011; Limem and Warner, 2005; Demirci et al., 2013).

In point-bonded fabrics, a further consideration is the fiber orientation web, geometric arrangement, and shape of the bonding points. The size and density of these thermal bonding points can be controlled to achieve desired properties such as absorbency, softness, and strength (Gharaei and Russell, 2022).

**Figure 5:**
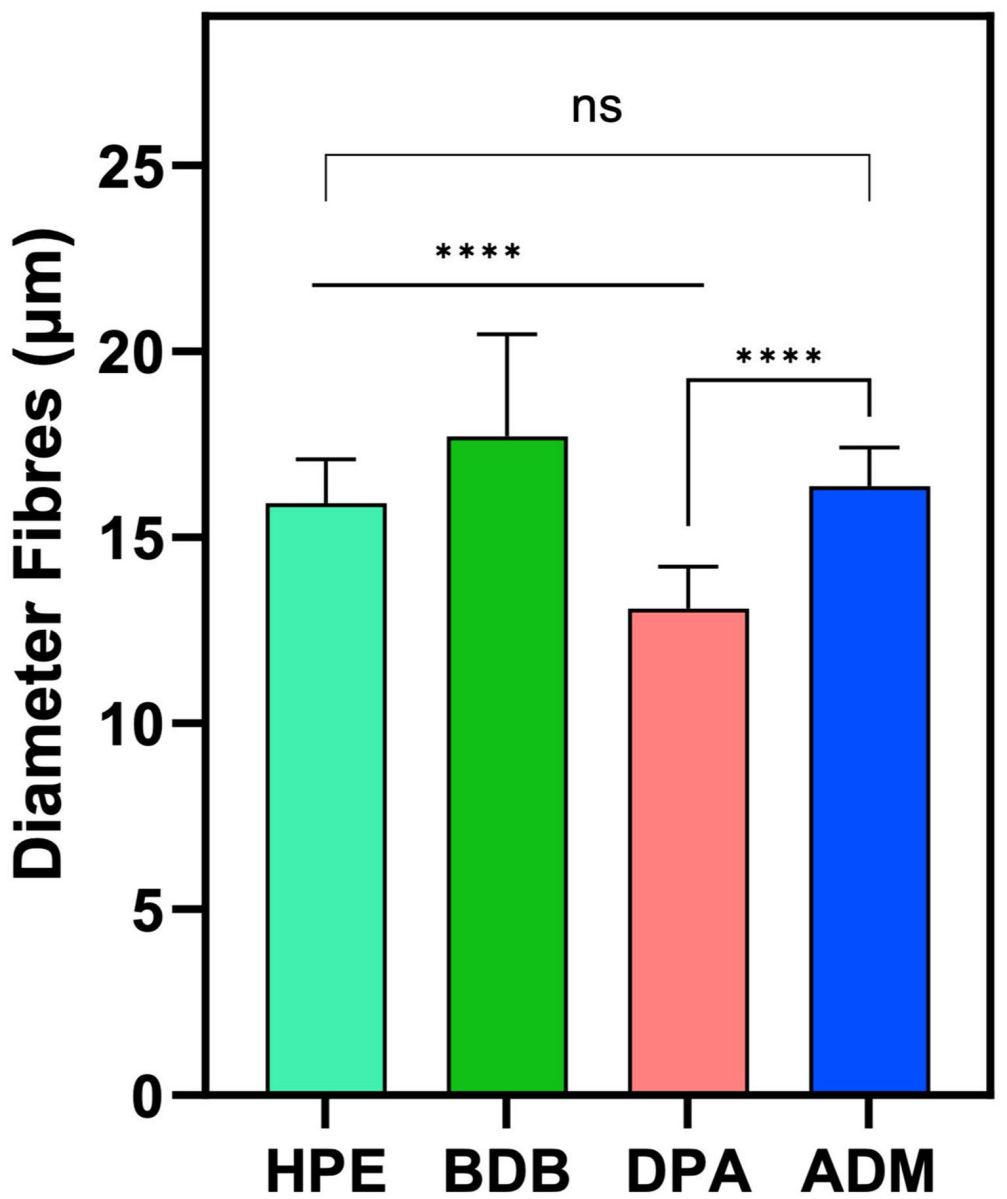
Diameter measurements were obtained from three independent samples (*N* = 3), with each sample measured in triplicate from different zones (*n* = 3). Statistical analysis indicated a significant difference (****p-value < 0.0001) between the indicated samples. However, no significant difference (ns) was observed between the HPE and ADM products.

Among the various products, the DPA product stands out due to its randomly arranged fibers, which are produced using the spunlace process —a technique commonly employed for manufacturing polyester nonwoven fibers (Niedziela et al., 2022). The DPA product has the smallest average fiber diameter of 13.05 μm across the entire set, as shown in Figure 5. The samples HPE, and ADM fibers have a size range between 15 and 16 μm, with no significant variations. The average diameter of BDB product measures 17.7 μm, which is slightly higher average diameter than the other samples.

### Mechanical properties

**Figure 6:**
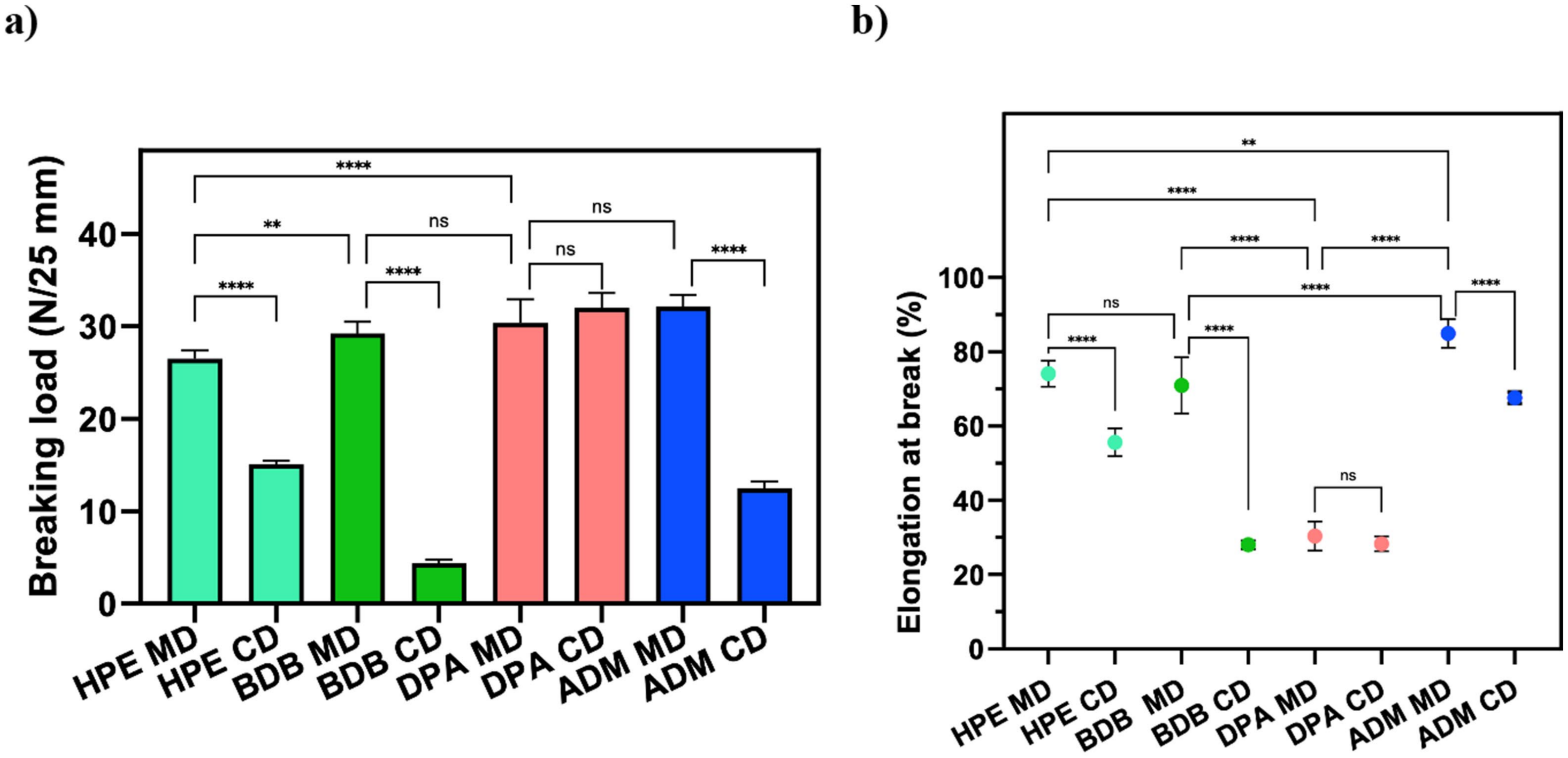
Mechanical properties of wet wipes: **(a)** Breaking point and **(b)** Elongation at the breaking point of commercial products. Statistical analysis indicates: ns (no significant differences) among samples, **p < 0.01, and ****p < 0.0001.

Samples were prepared and tested following the ISO 9073-3 standard guidelines (International Standard, 2023). Similar characteristics were observed in samples under tensile strain tests, as expected due to their composition. As anticipated, samples with comparable morphology showed similar breaking points and elongation values. Tensile tests were performed in both machine direction (MD) and cross direction (CD) to assess mechanical behavior. Figure 6a shows that HPE, BDB, and ADM products exhibited higher average breaking point values in the MD than those of the CD. This highlights the influence of fiber orientations and bonding points within the polyester nonwoven on their overall mechanical performance. The significant differences in the breaking load observed between MD and CD values can be attributed to fiber orientations and bonding points within polyester nonwoven materials (Leucker and Schubert, 2020). As fibers are aligned in MD, they provide greater enhanced resistance to the applied force, resulting in an increased breaking load. On the other hand, in CD there is less alignment among which results in the decreased mechanical resistance and low elongation at the breakage point (Leucker and Schubert, 2020; Demirci et al., 2013), as shown in Figure 6b. This was evident in the DPA sample results, where no significant differences were observed between the breaking loads and elongation values in both MD and CD due to the random arrangement of the fibers.

To further understand the elements that may contribute to the effectiveness of commercial wipes for healthcare disinfection, it is important to consider the chemical composition of the wipes. Additionally, compatibility of liquid disinfectants within the fibers, volume of disinfectant used, the presence or absence of other substances may also reduce surface tension and impact the wetting of the surface (Boyce, 2021; Song et al., 2022).

### Surface tension

**Figure 7:**
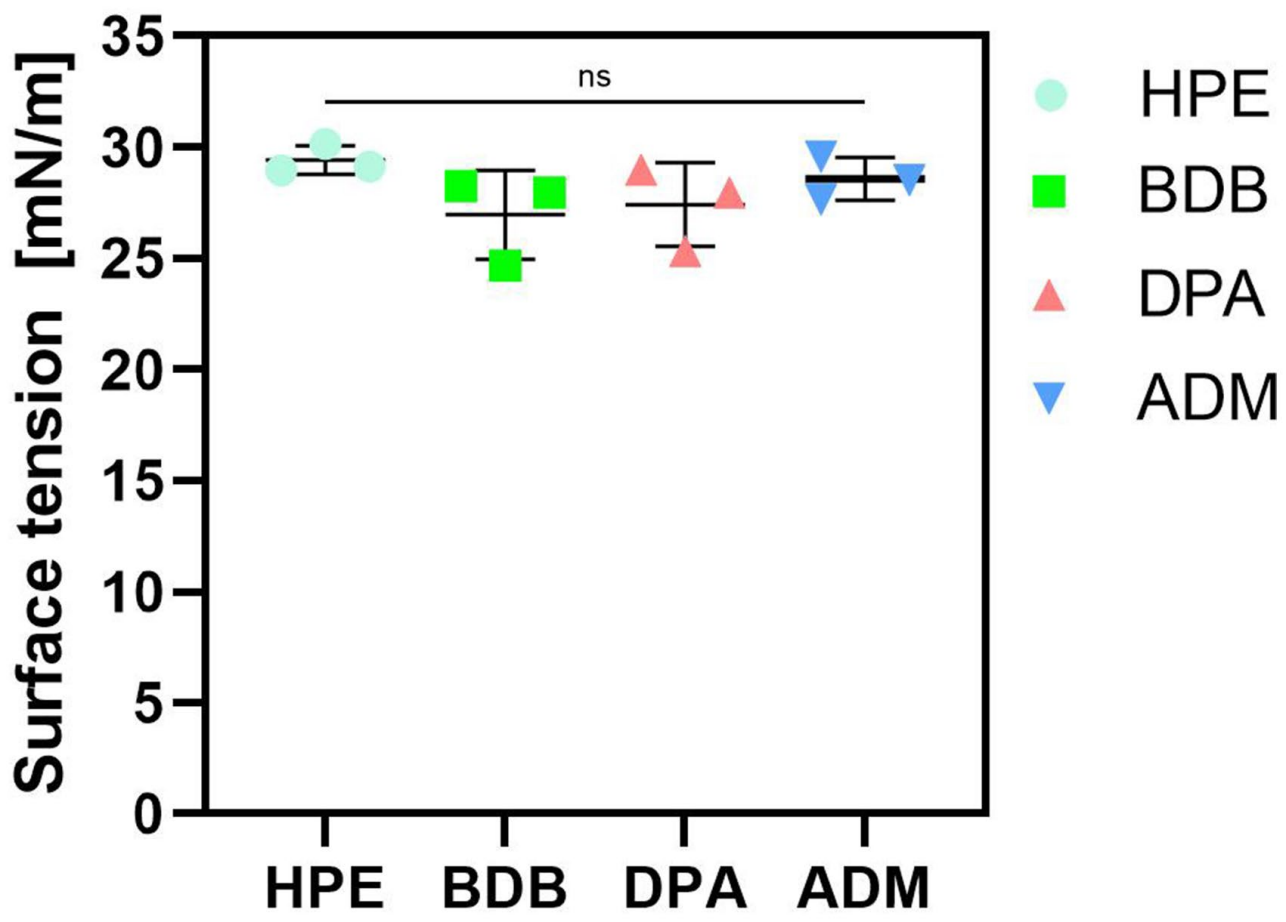
Measurements of surface tension of liquid disinfectants eluted. Three independent samples (*N* = 3) were tested. All samples labeled as “ns” (not significant) presented no significant differences among them.

Surface tension of a disinfectant plays a crucial role in its effectiveness and type of application. Lower surface tension promotes wide dispersibility across different surfaces, leading to an improved coverage and increased contact with pathogens. Values of surface tension obtained were consistent and aligned with the expectations of strong antimicrobial solutions (Figure 7), indicating that liquid disinfectant was designed to achieve effective killing of microorganisms while also possessing the properties for effective surface cleaning (Rao and Mulky, 2023; Kumari et al., 2023; Wojciechowski et al., 2023).

Commercial products typically include surfactants, along with active agents or biocides. These surfactants serve as enhancers of antimicrobial activity by reducing surface tension (Falk, 2019). This reduction in surface tension promotes the effectiveness of the active agents, leading to improved antimicrobial efficacy. Furthermore, fluids with low surface tension can effortlessly penetrate small cracks and gaps compared to those with high surface tension values. Moreover, disinfectant solutions possessing low surface tension have the ability to be absorbed onto surfaces of targeted organisms for effective elimination (Frobisher, 1927). Additionally, it is important to note that the effectiveness of disinfectant wipes may be influenced by factors such as fibers they are made from, compatibility with disinfectants, amount of disinfectant applied to each wipe, and presence of substances that reduce surface tension and that improve wetting (Tyski et al., 2021; Nkemngong et al., 2020).

### Disinfectant released from wet wipes made of non-biodegradable fibers

**Figure 8:**
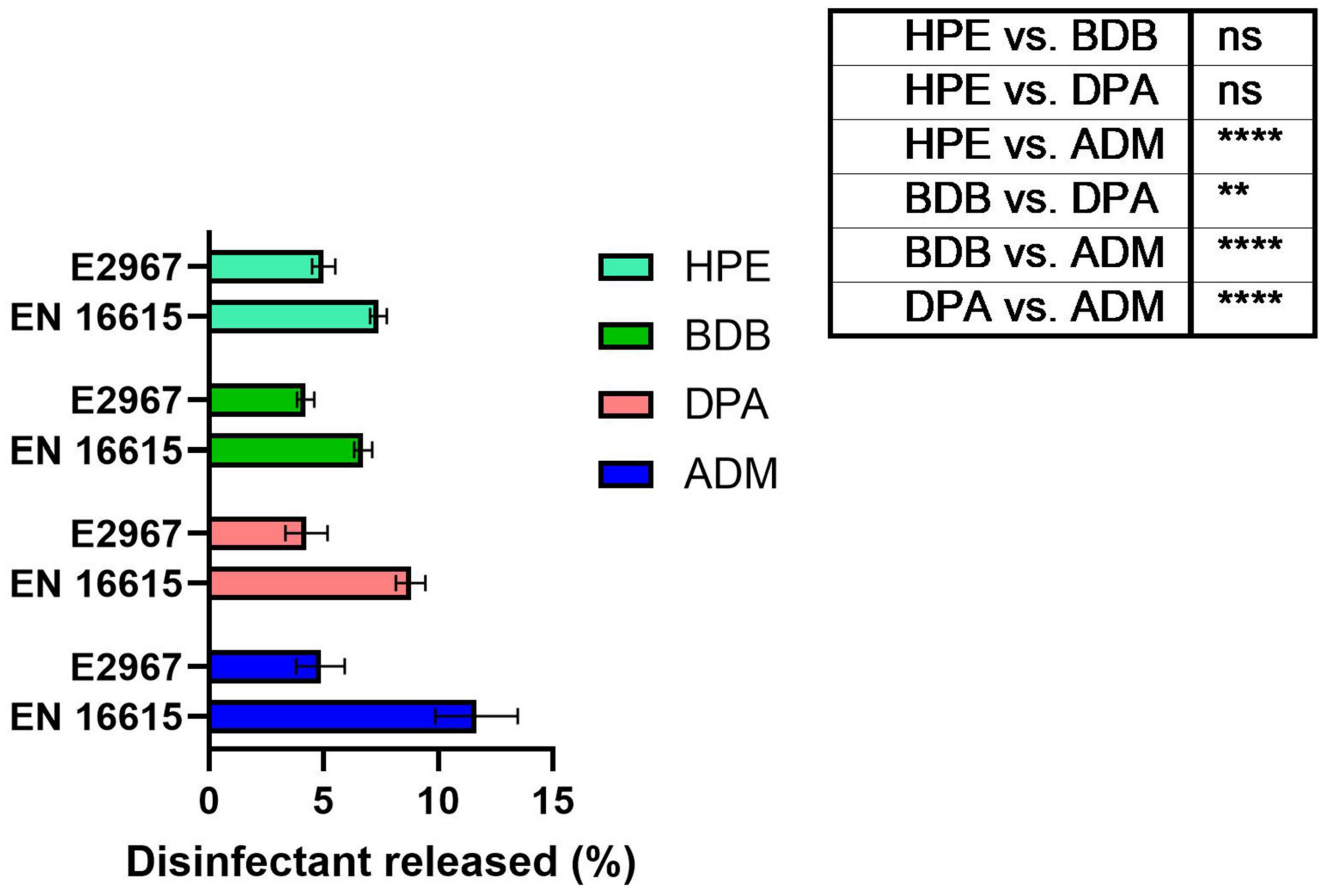
Percentage of liquid disinfectant released from the wet wipe products after usage under EN 16615 and ASTM E2967 on bacteria-free surfaces. *N* = 7 different samples tested. The sample set illustrates the *p*-values resulting from ANOVA tests, ****p value < 0.0001, ns: no significant difference.

In the design of wet wipe products, a critical factor to take into account is the amount of disinfectant released onto the surface during cleaning and disinfection (Ramm et al., 2015). It is widely recognized that achieving compatibility among different components of these products plays a fundamental role in ensuring high antimicrobial performance (Wesgate et al., 2018; Cave et al., 2021). Absorption and desorption capacities of non-biodegradable fibers have been extensively researched, indicating their ability to release higher quantities of liquid disinfectant than biodegradable fibers or cellulose-based ones. Particularly for wet wipes designed for sanitizing critical areas like hospitals and healthcare centers, optimizing liquid release from fibers becomes essential to meet standard criteria for public health applications (Wesgate et al., 2018; Song et al., 2022; Ramm et al., 2015). The amount of liquid disinfectant released from wet wipe products was measured according with the established standards designed to simulate bacterial testing scenarios. The percentage of disinfectant released in practical usage scenarios was determined by measuring the change in weight before and after applying a wipe to a bacteria free surface. As seen in Figure 8, the disinfectant release percentage of the commercial samples is situated within the range of 7–11%. This range is commonly observed for non-biodegradable samples made of polyester fibers (Jacobshagen et al., 2020). The product with the highest percentage of disinfectant release, as measured by the BS EN 16615 standard protocols, was the ADM product, with an average release of 11.6% relative to its initial mass. This result could be correlated to its performance in antibacterial tests, especially against harmful bacteria, as discussed later. Following the application of the Wiperator device, a consistent release of approximately 5% liquid disinfectant was observed across all samples, indicating negligible variations among them.

### Antibacterial evaluation using EN and ASTM standards

**Figure 9:**
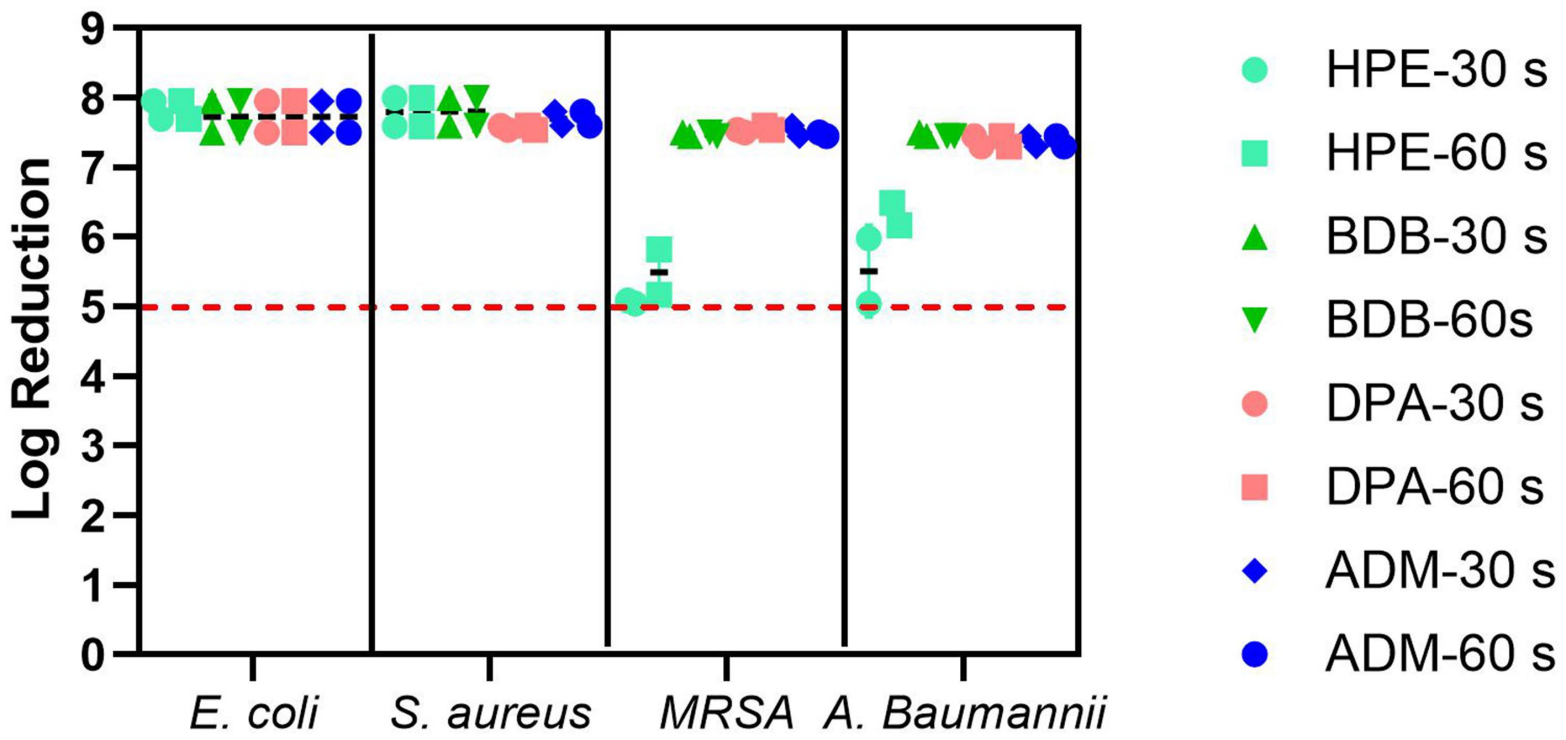
Bacterial reduction graph resulting from tests conducted according to EN 13727 standards. These tests involved *E. coli*, *S. aureus*, MRSA, and *A. baumannii*, assessing contact times of 30 and 60 s. The experiments were carried out in 2 independent times (*N* = 2) and in triplicates for each experiment (*n* = 3). The primary criteria, denoted by the red dotted line represents standard criteria (bacterial reduction > 5 Log).

Antibacterial evaluation is an essential process to assess the effectiveness of antibacterial coatings and materials. Liquid extracted or eluated from a wet wipe underwent evaluation with both 30 and 60 s of contact time, in accordance with EN regulatory guidelines. Each of the examined products fulfilled the minimum requirements for compliance with the standard, as illustrated in Figure 9, the red dotted line serves as the criterion for passing the standard, which is set at a 5 log reduction. Despite being tested under clean conditions, the HPE product narrowly met the reduction criteria for bacterial reduction when using disinfectant liquid against the tested resistant bacteria. This specific product employed a disinfectant formulation containing hydrogen peroxide as bioactive agent. This observation was noted during testing against MRSA using both contact times and similarly when testing against *A. baumannii* with a 30-s contact time. Both bacterial strains are a significant public health issue nowadays due to antibiotic resistance and the severity of diseases caused by these challenging-to-treat infections (Ren and Palmer Lauren, 2023; Hasanpour et al., 2023). Bacteria can exhibit natural tolerance or reduced susceptibility to biocides due to inherent physiological factors. Microbial adaptations that enhance survival in the presence of biocides may also enable resistance to other compounds, such as antibiotics, a phenomenon known as cross-resistance. Furthermore, resistance mechanisms to both biocides and antibiotics can be located within mobile genetic elements, allowing the selection of one resistance trait to potentially co-select for another through the propagation of the entire genetic element, a process referred to as co-resistance. These factors empower them to survive, and in some instances, thrive in solutions containing these substances (Fox et al., 2021).

**Figure 10:**
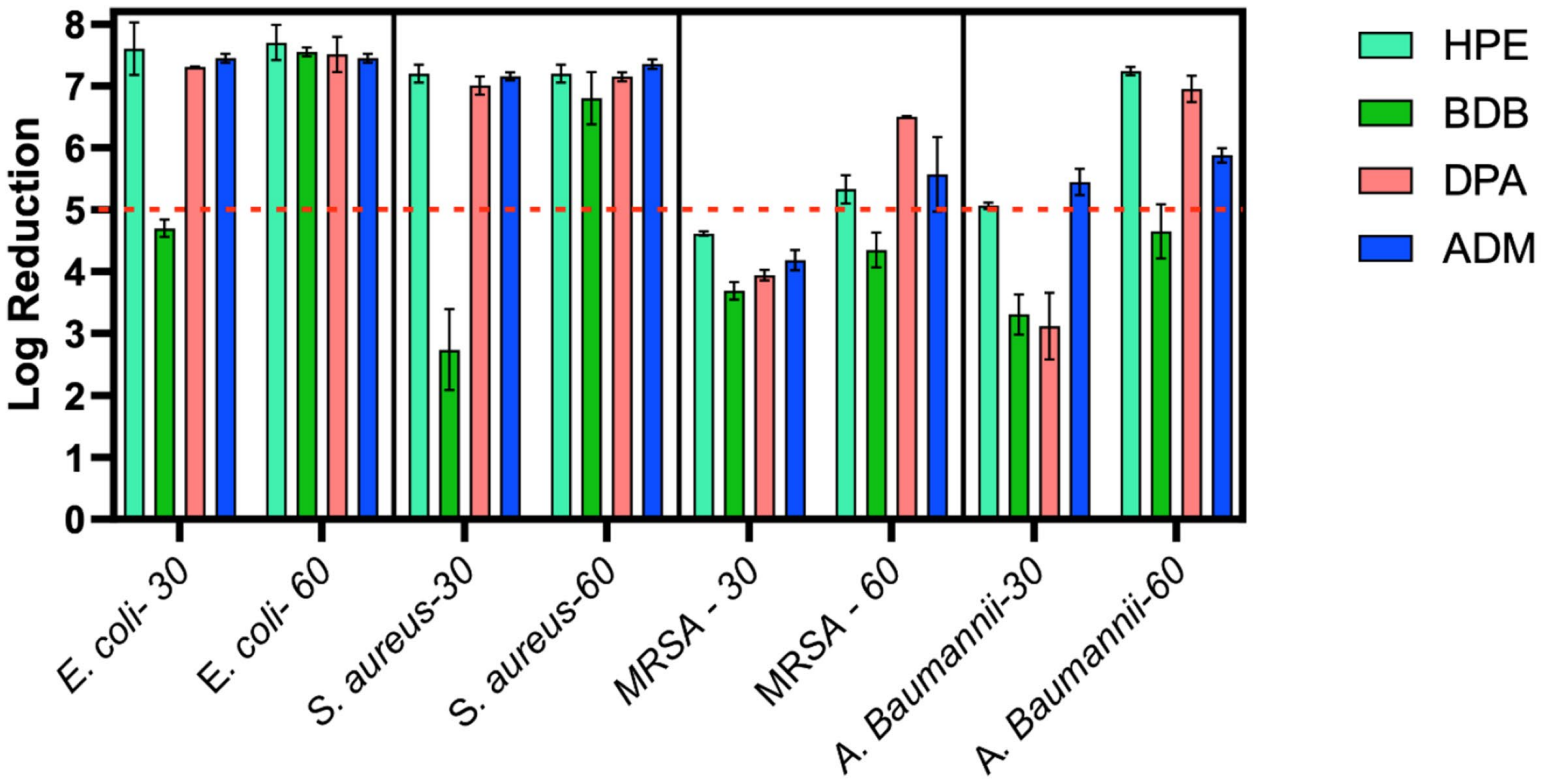
Bacterial reduction graph resulting from tests conducted according to EN 16615 standards. These tests involved *E. coli*, *S. aureus*, MRSA, and *A. baumannii*, assessing contact times of 30 and 60 s. Experiments were carried out in 2 independent times (*N* = 2) and in duplicate for each experiment (*n* = 2). Main criteria denoted by the red dotted line (Bacterial reduction > 5 Log).

**Figure 11:**
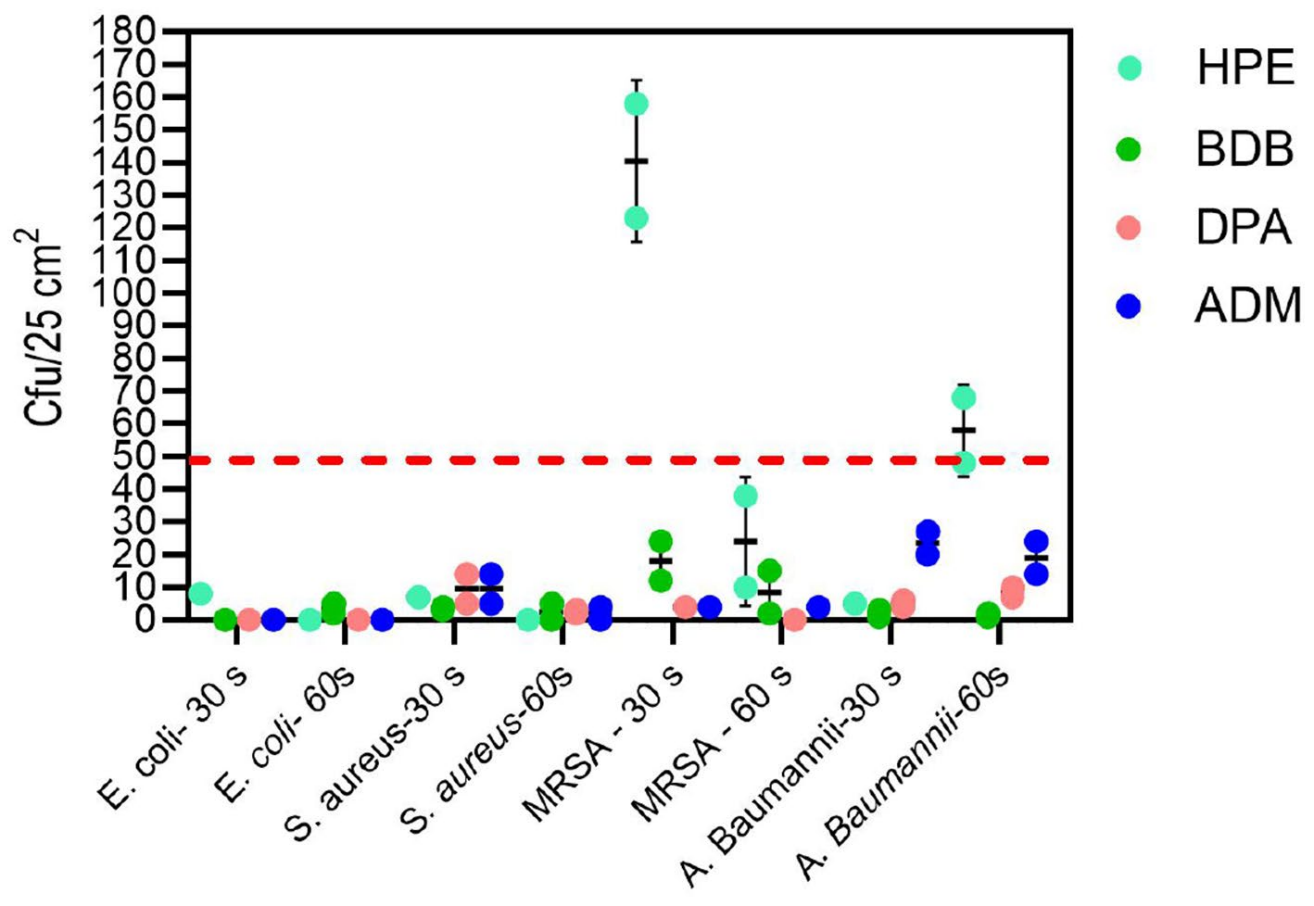
Analysis of bacterial spread according to the second criteria of EN 16615 tests, measured in CFU/25 cm^2^ from non-inoculated surfaces F2 to F4 on the PVC surface (Figure 2) for each bacterial strain at 30 and 60 s of contact time. Experiments were conducted independently twice (*N* = 2), with each experiment performed in duplicate (*n* = 2). The dotted line shows the criteria average from F2 to F4 < 50 CFU.

The remaining products complied with the criteria specified in the BS EN 13727 standard, meaning they were able to achieve a 5-log reduction against the bacteria tested. Examining the eluate from wet wipe products holds the potential to establish a link between the active biocidal agents released onto the intended disinfection surface. Based on data obtained from the 4-field tests described in the BS EN 16615, manufacturers can demonstrate the efficiency of their products in eliminating harmful microorganisms. The tests conducted following the BS EN 16615 standard have revealed that the BDB product, with a contact time of 60 s, did not meet the criteria for effectiveness against MRSA and *A. baumannii,* as shown in Figure 10. This raises the level of concern, as the tests were conducted under clean conditions, which are typically considered the least challenging conditions for meeting the required standards. Acknowledging the challenges associated with eliminating these bacterial strains, it is imperative to note their common presence in healthcare facilities. This underscores the importance of employing wet wipes for surface disinfection to effectively eradicate them. HPE, DPA and ADM products successfully meet the testing criteria, using 60 s of contact time against all tested bacterial strains. These results imply that these samples fulfill both criteria: bacterial reduction > 5 Log in inoculated surface (see Table 2) and average CFU < 50 from non-inoculated surfaces. The HPE product did not meet the second criteria when tested against MRSA and *A. baumannii* with 30-s contact time (Figure 11). This correlated with the fact that it also failed the main criteria test in inoculated surface reduction, which may contribute to the spread of these bacteria in the remaining clean fields, without prior bacterial inoculation.

**Table 2:** Summary results of log reduction values from EN 16615 using commercial products at 30 and 60 s of contact time.

F_1_ - Log R	HPE		BDB		DPA		ADM	
*E. coli*-30 s	7.9	7.3	4.6	4.8	7.31	7.3	7.5	7.4
*E. coli*-60 s	7.9	7.5	7.5	7.6	7.31	7.71	7.5	7.4
*S. aureus*-30 s	7.1	7.3	3.2	2.28	7.11	6.9	7.11	7.2
*S. aureus*-60 s	7.1	7.3	7.1	6.5	7.2	7.1	7.41	7.3
MRSA-30 s	4.64	4.59	3.59	3.79	3.88	4	4.07	4.3
MRSA-60 s	5.49	5.17	4.55	4.15	6.51	6.49	5.15	6
*A. baumannii*-30 s	5.1	5.03	3.08	3.54	2.74	3.5	5.6	5.3
*A. baumannii*-60 s	7.19	7.29	4.34	4.96	6.8	7.1	5.8	5.96

**Figure 12:**
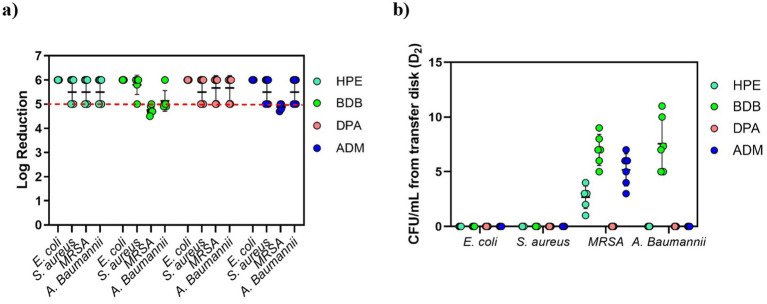
**(a)** Bacterial reduction graph resulting from tests conducted according to ASTM E2967-15 standard. These tests involved *E. coli*, *S. aureus*, MRSA, and *A. baumannii*, assessing contact time of 60 s. The main criteria denoted by the red dotted line, represents a > 4 log reduction. **(b)** CFU/mL from the transfer Disk, D2. Experiments were carried out in 2 independent times (*N* = 2) and in triplicate for each experiment (*n* = 3).

Another test utilized was the ASTM E2967-15 test, which also evaluates the efficacy of wet wipes against bacteria. As expected, all specimens passed the test successfully, showing a reduction > 5 log from the inoculated disk due to the orbital movement employed in the wiping procedure, as shown in Figure 12a. No significant amount of CFUs was detected on TSA plates from commercial products in D2 (transfer disk) (Figure 12b).

## Discussion

### Evaluation of antibacterial efficacy using the three different methods

The eluate tests confirm that all products have successfully met the prescribed disinfectant standard. However, it is important to recognize the limitations of BS EN 13727 for assessing disinfectant wet wipe products, especially regarding their intended usage. This remains true even when examining eluates derived from such wipes. Claims based on tests without wiping actions may not accurately reflect real-life situations (Tarka et al., 2019). Dynamic tests using the wet wipes provide a more precise assessment of the practical effectiveness of disinfection procedures, ensuring the elimination of microorganisms (Centeleghe et al., 2022).

It is evident that at short contact times—typically employed by commercial brands for product promotion—achieved values for bacterial reduction that were the lowest, failing to meet the criteria outlined by BS EN 16615. At the 30-s contact time, all products failed to meet the criteria set by the BS EN standard for one or more of the bacterial strains evaluated at clean conditions. During the other short contact times of testing, BDB product performed the lowest efficacy, failing against all tested strains including *E. coli* and *S. aureus*. Of particular concern were the results for the MRSA strain, as none of the products met the minimum criteria specified in BS EN 16615. Additionally, only ADM product, which contained a mixture of Quats and a diamine product, met both criteria against *A. baumannii* at the 30-s contact time. These findings clearly indicate that it is crucial for commercial brands to reconsider their practice of testing wet wipes with a contact time of only 30 s or less, to comply with the regulatory protocols, despite BS EN 16615 specifying a minimum contact time of 60 s. A critical factor for consideration is the volume of disinfectant released onto the target surface. The results of this study indicate a direct correlation between this variable and antibacterial efficacy. Notably, the ADM product, which exhibited superior performance due to meet all criteria of BS EN standard, achieved a high release percentage on the surface designated for cleaning or disinfection relative to its initial mass. This finding suggests that the initial disinfectant dosing could be a crucial factor in developing and optimizing new disinfectant formulations. In accordance with the bacterial transmission assessment protocol outlined in EN 16615, no bacterial transfer was observed from Field 2 (F2) to Field 4 (F4) (refer to Figure 2) following a 60-s contact period under clean conditions. However, when the HPE product was applied with shorter contact time, the transfer of both MRSA and *A. baumannii* to non-inoculated surfaces was detected. MRSA transmission to clean surfaces (D2) was minimal when using the ASTM standard (ASTM International, 2015) method of orbital movement for 5 s on contaminated stainless-steel surfaces. However, the infectious dose for MRSA is relatively low. Dancer (2014) reported that only 4 CFU were required for infection, and the pathogen can survive on surfaces for up to 7 days, increasing infection risk. The World Health Organization recognizes the threat posed by MRSA and other antibiotic-resistant pathogens. As part of broader infection prevention and control strategies, effective cleaning and disinfection of surfaces in healthcare settings are crucial to prevent the spread of these organisms (Severn and Horswill, 2023). This strain exhibits high adaptability and the potential to cause life-threatening diseases. Therefore, there is a critical need for the development of more effective products capable of efficiently mitigating and eradicating bacterial spread in healthcare settings (Craft et al., 2019).

In Jacobshagen et al., 2020 discussed the reduction of bacterial transfer when using the Wiperator™ and following the guidelines outlined in BS EN 16615. In general, there is a tendency to achieve a greater reduction in the number of CFUs when using Wiperator™ method. This can be attributed to factors such as increased pressure and longer wiping time. Additionally, the dynamic wiping motion aids in this effect by applying shear and compressive forces that detach bacteria from surfaces and transfer them onto the wipe (Edwards et al., 2019). However, it should be noted that these forces are not as effective when the wet wipe is moved horizontally during tests such as the 4-field test.

## Conclusion

The development of effective disinfectant products for healthcare settings requires a thorough evaluation of material compatibility, surface tension, and the release of disinfectants onto target surfaces. Optimizing these parameters is important for enhancing the performance of disinfectant wipes and potentially reducing the risk of healthcare-associated infections. To accurately assess the antibacterial efficacy of wet wipes, standardized tests such as EN 16615 are recommended, as they ensure reliable results for evaluating the effectiveness of disinfectant wipes against bacteria. The BDB product exhibited relatively diminished efficacy compared to other samples when evaluated against EN standards criteria commonly used for hospital disinfection claims. Its performance was particularly suboptimal during short contact times (<60 s), failing to achieve required efficacy against tested strains, including *E. coli* and *S. aureus*. Notably, all products, did not fully meet EN 16615 minimum criteria for MRSA reduction, highlighting an area necessitating further research and development in disinfectant formulations for healthcare settings. The HPE, DPA, and ADM products demonstrated effective bacterial reduction within 60 s. Notably, ADM was the only product to meet criteria of BS EN 16615, achieving a reduction > 5 Log and preventing the spread of *A. baumannii* from contaminated to clean surfaces within short contact times. The superior antibacterial efficacy exhibited by the ADM product can be attributed to the positive correlation between its active disinfectant composition and optimal release volume, highlighting the significance of formulation in maximizing antimicrobial performance. These findings provide a foundation for enhancing disinfectant wipe design by revealing the critical role of material properties and disinfectant composition in determining effectiveness. This insight reveals opportunities to optimize formulations, ultimately leading to improved microbial reduction and more effective infection control strategies in healthcare environments.

## Data Availability

The raw data supporting the conclusions of this article will be made available by the authors, without undue reservation.
